# TAF7 accumulates in the cytoplasm during cellular transformation and engages STAT3, WASH, and CCT

**DOI:** 10.3389/fcell.2026.1855716

**Published:** 2026-07-08

**Authors:** Dan Cheng, Elizabeth McManus, Ryan M. Young, Thomas Oellerich, Bjorn Haupl, Daniel D. Billadeau, Hua Tan, Laura Elnitski, Dinah S. Singer

**Affiliations:** 1 Experimental Immunology Branch, Center for Cancer Research, National Cancer Institute, NIH, Bethesda, MD, United States; 2 Department of Molecular Biology, Princeton University, Princeton, NJ, United States; 3 Lymphoid Malignancies Branch, Center for Cancer Research, National Cancer Institute, NIH, Bethesda, MD, United States; 4 Department of Medicine, Hematology and Oncology, University Hospital, Goethe University Frankfurt, Frankfurt am Main, Frankfurt, Germany; 5 Division of Oncology Research, Mayo Clinic College of Medicine, Rochester, MN, United States; 6 Translational and Functional Genomics Branch, National Human Genome Research Institute, NIH, Bethesda, MD, United States

**Keywords:** CCT, cytoplasmic TAF7, oncogenesis, STAT3, transformation, WASH

## Abstract

TAF7, a core subunit of the general transcription factor TFIID, regulates transcription and additionally functions as an RNA chaperone that directs nascent nuclear RNAs to cytoplasmic polysomes, thereby influencing protein synthesis. Here, we identify a set of novel cytoplasmic interactors and a previously unappreciated cytoplasmic role for TAF7 in oncogenesis. BioID-mediated proximity labeling identified STAT3 and the CCT and WASH complexes as prominent cytoplasmic interactors of TAF7. These interactions were validated by co-immunoprecipitation and proximity ligation assays. We further showed that cytoplasmic TAF7 levels increased during oncogenesis in proportion to cancer cell pathogenicity. Notably, TAF7 engagement with STAT3, CCT and WASH complexes is markedly enhanced during cellular transformation, consistent with their functions in cancer progression. Together, these findings reveal a cytoplasmic TAF7 interactome and implicate elevated cytoplasmic TAF7 as associated with oncogenesis.

## Introduction

Maintaining homeostasis in cells requires the integration of multiple cellular processes including transcription, RNA export to polysomes, proper folding of nascent proteins and proper sorting of proteins to endosomes and other cellular compartments. All these activities must be coordinated to support cell proliferation and survival. While considerable attention has been focused on the mechanisms regulating each of these functions, studies are only now beginning to examine how they are coordinated. We have reported that the transcription factor TATA box–binding protein (TBP)–associated factor 7 (TAF7), an essential transcription factor, coordinates transcription initiation, elongation, RNA transport to cytoplasmic polysomes and translation (Gegonne et al., 2013; Cheng et al., 2021).

TAF7 plays a critical role in transcription, regulating initiation, pause release, early elongation and productive elongation through its interactions with, and inhibition of, the enzymatic activities necessary for each step (Gegonne et al., 2008; Devaiah and Singer, 2012). Thus, TAF7 binds to the TFIID component TAF1, inhibiting the TAF1 acetyl transferase activity required for transcription initiation (Mizzen et al., 1996; Weissman et al., 1998; Gegonne et al., 2001). TAF7 then binds to and inhibits the kinase activities of CDK7/TFIIH, BRD4, CDK9/PTEFb (Gegonne et al., 2006; Gegonne et al., 2008; Devaiah and Singer, 2012). During productive elongation, TAF7 travels with the elongation complex, where it binds to a large spectrum of nascent RNA species at consensus CUG motifs in the 3′ UTR region of its target transcripts (Cheng et al., 2021). Remarkably, TAF7 transports bound RNA from the nucleus to the cytoplasm in an exportin-dependent process and delivers the RNA to cytoplasmic polysomes (Cheng et al., 2021). Disruption of TAF7 binding to its target RNAs or depletion of cytoplasmic TAF7 reduces protein synthesis globally (Cheng et al., 2021). In this way, TAF7 coordinates transcription in the nucleus with translation in the cytoplasm.

Beyond coordination of transcription and translation, cellular homeostasis requires responses to extracellular signaling, proper protein folding and protein segregation within cellular compartments. Mediators of each of these activities have been identified. Among the best characterized responses to extracellular signaling is that of the Src pathway which is activated by cytokines and growth factors and results in the phosphorylation of transcription factor, STAT3, and its translocation into the nucleus, where it activates target gene expression (Turkson et al., 1998). Downstream of transcriptional regulation and translation, proper protein folding is achieved by a variety of chaperones that are known to ensure co-translational folding of nascent proteins (Frydman, 2001). Among these is the CCT (also known as TriC) complex, consisting of two rings of eight subunits each (Lopez et al., 2015). CCT folds actin and tubulin co-translationally but also interacts with other mature proteins post-translationally (Lopez et al., 2015). Protein segregation and endosomal sorting is mediated in part by the WASH complex which is one of several different Wiskott-Aldrich syndrome proteins (WASP) family members that are responsible for actin remodeling (Alekhina et al., 2017). The WASH complex activates actin filament assembly and regulates endosomal sorting (Derivery et al., 2009; Gomez and Billadeau, 2009; Seaman et al., 2013).

Our finding that TAF7 functions to transport RNA to the cytoplasm and to regulate translation led us to speculate that it has additional cytoplasmic functions. Remarkably, we find that the WASH complex, the CCT complex and STAT3 are among the major cytoplasmic TAF7 interactors. All three of these factors–STAT3, CCT, WASH–play important roles in the maintenance of cellular homeostasis. During cellular transformation, normal cellular homeostasis is perturbed to respond to the requirements of the cancer cell (Hanahan, 2022). Many transcriptional, translational, metabolic and signaling pathways are constitutively upregulated. The finding that TAF7 interacts with STAT3, CCT, WASH–all of which have been shown to play a role in cancer (Yu et al., 2009; Zech et al., 2011; Deng et al., 2015; Huang et al., 2017; Vallin and Grantham, 2019; Zou et al., 2020; Ghozlan et al., 2022; Zeng et al., 2024) - led us to ask whether the levels of TAF7 in the cytoplasm correlate with the disruptions in cellular homeostasis associated with transformed cancer cells. As predicted, the relative levels of cytoplasmic TAF7 increase with cellular transformation and pathogenicity, leading to increased interactions with the WASH complex, the CCT complex and STAT3. Together, these findings indicate a potential role for TAF7 in coordinating homeostasis in normal cells and in cancer.

## Materials and methods

### Cell culture and cell transformation

Hela cells and MDA-MB-231 cells were cultured in DMEM medium supplemented with 10% fetal bovine serum. MCF10A cells were grown and maintained as described by Dr. Joan Brugge’s lab (Debnath et al., 2003). The premalignant MCF10AT1k.cl2 (“M2”), the early malignant MCF10Ca1h (“M3”) and the highly malignant, metastatic MCF10Ca1a.cl1 (“M4”) cell lines were gift from Dr. Binwu Tang. M2 cells were cultured in the same medium as MCF10 cells. M3 and M4 cells were cultured DMEM/F12 medium supplemented with 5% horse serum and 1% penicillin-streptomycin as described (Tang et al., 2003). MCF10A-ER-Src cells were kindly provided by Dr. Kevin Struhl and cultured in DMEM/F12 medium supplemented with 5% charcoal-stripped fetal bovine serum and other growth factors as described previously (Iliopoulos et al., 2009). The transformation of MCF10A-ER-Src cells was triggered by 1 µM tamoxifen (TAM) (Millipore Sigma) treatment for 24 h. T-ALL cells were kindly provided by Dr. Dean Felsher and cultured in RPMI medium supplemented with 5% horse serum and 1% penicillin-streptomycin. Doxycycline at 20 ng/mL was used to turn off MYC expressions. All cells were maintained at 37 °C in a humidified atmosphere containing 5% CO_2_.

### Antibodies

The antibodies used in this study were obtained from various sources: anti-TAF7 (TAF7 monoclonal antibody M01, clone 2C5, Abnova); anti-TBP (ab63766, Abcam); anti–β-tubulin (ab6046, Abcam); anti-Flag (clone M2, Millipore Sigma); anti-STAT3 (ab68153, Abcam); anti-CCT2 (ab92746, Abcam); anti-CCT5 (ab225876, Abcam); anti-phospho-STAT3 (9145L, Cell Signaling Technology); anti-Actin (sc-47778, Santa Cruz); anti-WASHC1 and anti-WASHC2 antibodies were described previously (Gomez and Billadeau, 2009).

### Nuclear and cytoplasmic fractionation

The cells were washed using PBS and collected by a brief centrifugation. Then cells were lysed with ice-cold lysis buffer [10 mM NaCI, 3 mM MgCl_2_, 10 mM Tris-HCl (pH 7.4), 0.5% NP-40] for 30 s, following by centrifuging the cell lysates at 17,000 g for 30 s to pellet the nuclei. The supernatant was collected as cytoplasmic extracts. The nuclei were subjected to further lysis using ice-cold nuclear lysis buffer [20 mM HEPES (pH 7.9), 400 mM NaCI, 1 mM EDTA, 10% glycerol], incubated for 2 h at 4 °C with rotation. After centrifugation at 17,000 g for 5 min, the supernatant was collected as nuclear extracts.

### Immunoprecipitation (IP) and immunoblotting

For IP, cell extracts (antigen) were incubated with antibody or the control mouse IgG/rabbit IgG overnight at 4 °C with gentle mixing, and then Protein G agarose slurry (Thermo Fisher Scientific) was added to the antigen-antibody/antigen-control IgG complex. The reaction was incubated for 2 h at 4 °C with gentle mixing. After that, the complex-bound resins were washed four times with ice-cold IP buffer [25 mM Tris- HCl (pH 7.4), 150 mM NaCI]. The immunoprecipitants were eluted by heating in 2×SDS loading buffer at 70 °C for 10 min.

For immunoblotting, proteins in samples were separated on SDS-PAGE and transferred onto nitrocellulose membranes (GE Healthcare). After blocking with 5% skim milk in TBST, the blots were incubated with the primary antibodies overnight at 4 °C. Protein detections were performed using secondary antibodies IRDye®800CM or IRDye®680RD (LI-COR Biosciences), and signals were developed with Odyssey membrane imagers (LI-COR Biosciences).

### Immunofluorescence and proximity ligation assay (PLA)

Immunofluorescence staining was performed as previously described (Cheng et al., 2021). Briefly, cells were fixed in 4% paraformaldehyde for 10 min, washed three times with PBS, and permeabilized with 0.5% Triton X-100. After that, cells were pre-blocked in 5% goat serum and 1% BSA for 1 h at room temperature, followed by incubating with primary antibodies overnight at 4 °C. The primary antibodies were detected with fluorescently conjugated secondary antibodies (Invitrogen). The staining was mounted in ProLong® Gold antifade reagent with DAPI (Thermo Fisher Scientific). Cells were then observed with Zeiss LSM880 Multi-photon Microscope.

The PLA was conducted using the Duolink® *In Situ* PLA Red Starter Kit Mouse/Rabbit (MilliporeSigma) according to the manufacturer’s protocol.

Quantitative images analysis was performed using custom macros in ImageJ. Multiple fields of view were analyzed for each condition, and measurements from all cells within a given field were averaged. Each data point shown in the figures represents the average value from one field of view. Overall, more than 200 cells were analyzed for each condition.

### BioID

BioID2 (Addgene 80,899) with an 8X linker of GSGGG and a SnaBI site was amplified by PCR with the following primers.

#### BioID2 Fwd

AAT​TCG​AAT​TCC​TGA​AGG​GCC​ACC​atg​tat​ccc​tat​gat​gtg​cca​gac​tat​gct​TTC​AAG​AAC​CTG​ATC​TGG​CTG​AAG​G.

#### BioID2 Rev

cgc​cgg​ccc​tcg​agg​tac​gta​cta​AGC​GCT​TCT​TCT​CAG​GCT​GAA​C.

The PCR fragment was purified and cloned into the StuI site in the MCS of pBMN-LYT2. The resulting BioID2-8Xlinker-pBMN-LYT2 vector permitted the addition of a BioID2-linker to the amino terminus of the TAF7 gene by inserting it at the SnaBI site using Gibson cloning (New England Biolabs). Synthetic gene fragments of TAF7 were cloned into this vector.

Resultant BioID2 constructs were packaged into retrovirus using 293 T cells with helper plasmids pHIT60 and pHIT/EA6x3* in a 2:1:1 ratio in Optimem (Gibco) with Trans-IT 293 (Mirus) as previously described (Phelan et al., 2018). Infected cells were purified with LYT2 magnetic beads (Invitrogen). Cells were then grown in SILAC media containing amino acids labeled with stable isotopes of arginine and lysine, for 2 weeks and then expanded to 50 × 10^6^ cells. Biotin (MilliporeSigma) was added to a final concentration of 50 μM to transduced cells 16 h prior to lysis. Cells were then lysed at 2.5 × 10^7^ cells/mL in modified RIPA buffer (1% NP-40, 0.5% deoxycholate, 50 mM Tris, pH 7.5, 150 mM NaCl, 1 mM Na_3_VO_4_, 5 mM NaF, 1 mM AEBSF) for 10 min on ice. Lysates were pre-cleared by centrifugation at 14,000xg for 20 min at 4 °C and incubated with 35 μL of pre-washed streptavidin agarose beads (Thermo Fisher Scientific) for 2 h at 4 °C with rotation. Reactions were then washed four times with RIPA buffer and solubilized with 4X LDS sample buffer (Invitrogen) with 1% Nupage reducing agent (Invitrogen), and boiled for 5 min.

For MS analysis, proteins were separated by gel electrophoresis (4%–12% NuPAGE Bis-Tris Gel, Invitrogen), and the entire lane of a Coomassie blue-stained gel was cut into slices, which were processed as described previously (Oellerich et al., 2013). After tryptic digestion of the proteins the resulting peptides in the gel matrix were resuspended in sample loading buffer (2% acetonitrile and 0.05% trifluoroacetic acid) and were analyzed LC/MS on an UltiMate 3,000 RSLCnano HPLC system (Thermo Fisher Scientific) coupled online to a Q Exactive HF mass spectrometer (Thermo Fisher Scientific) as previously described (Phelan et al., 2018).

MS data analysis was performed using the software MaxQuant (version 1.6.0.17) by matching the mass spectra to the UniProtKB/Swiss-Prot human database. Minimal peptide length was set to seven amino acids, with a maximum of two missed cleavages. The false discovery rate (FDR) was set to 1% on both the peptide and the protein level using a forward-and-reverse concatenated decoy database approach. For SILAC quantification, multiplicity was set to two or three for double (Lys+0/Arg+0, Lys+8/Arg+10) or triple (Lys+0/Arg+0, Lys+4/Arg+6, Lys+8/Arg+10) labeling, respectively. At least two ratio counts were required for peptide quantification. The “re-quantify” option of MaxQuant was enabled. Data are deposited on MassIVE DB with the identifier PXD080431.

### Survival analysis of TCGA-UCEC patients by TAF7 mutation status

Statistical significance was determined by the log-rank test. Somatic mutation data and clinical metadata for the Uterine Corpus Endometrial Carcinoma (UCEC) cohort were retrieved from The Cancer Genome Atlas (TCGA) data portal (https://portal.gdc.cancer.gov/). All statistical analyses were performed on R version 4.5.2. Due to the small number of patients with TAF7 mutation, a P-value <0.1 was deemed significant.

### Statistical analysis

The data are presented as means ± standard deviation (S.D.) of at least three independent experiments. The “p” values indicate statistical significance, which is obtained using two-tailed, unpaired student’s t-test.

## Results

### Cytoplasmic TAF7 interacts with proteins involved in signaling, protein folding and endosomal sorting

**FIGURE 1:**
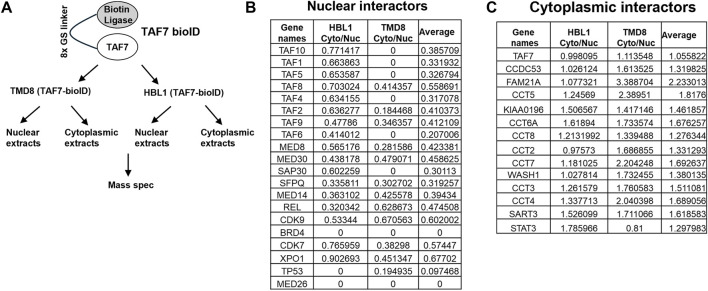
BioID probing identifies STAT3, WASH and CCT as TAF7 interactors in the cytoplasm. **(A)** Schema for bioID analysis of TAF7 interactors. TAF7 DNA was linked to the birA biotin ligase and transfected into TMD8 and HBL-1 B lymphoblastic cell lines. Following culture with biotin, cells were fractionated and extracts subjected to streptavidin capture, followed by mass spec, as detailed in Experimental procedures. **(B)** List of top nuclear TAF7 interactors identified in both cell types. The cytoplasmic/nuclear ratio is calculated from the mass spec counts. **(C)** List of top cytoplasmic TAF7 interactors identified in both cell types. The cytoplasmic/nuclear ratio is calculated from the mass spec counts.

We have previously reported that TAF7, a component of the nuclear general transcription factor TFIID, also functions as an RNA chaperone, shuttling newly synthesized transcripts from the nucleus to cytoplasmic polysomes (Cheng et al., 2021). To explore possible additional roles of cytoplasmic TAF7, we asked whether TAF7 interacts with other cytoplasmic proteins. To this end, we used the bioID assay, which labels proteins in proximity to TAF7 with biotin, allowing them to be purified with avidin and characterized (Yang et al., 2022). Briefly, a construct in which TAF7 was ligated with the biotin ligase, BirA, was transfected into two independent B-cell lymphoma lines, TMD8 and HBL-1. Following growth in the presence of biotin, cells were fractionated into nuclear and cytoplasmic lysates; biotin labeled proteins were captured with avidin and subjected to mass spectrometry (MS) analysis (Figure 1A).

MS analysis of biotin-labeled nuclear proteins identified the expected TAF7 interactors (Figure 1B; Supplementary Figures S1A,C). Namely, among the proteins were many components of TFIID (TAF1,2,4,5,6,8,9,10). In addition, the transcription factors BRD4, CDK7 and CDK9, which we previously reported to interact with TAF7, were detected (Gegonne et al., 2008; Devaiah and Singer, 2012). Other studies have reported that TAF7 interacts with the MED26 component of the Mediator complex (Takahashi et al., 2015). Accordingly, several Mediator components were detected in the mass spec analysis (Figure 1B; Supplementary Figures S1A,C). Novel interactors were also identified, including the NFκB component, Rel, and the tumor suppressor protein, TP53 (Figure 1B; Supplementary Figures S1A,C).

Importantly, a number of novel cytoplasmic TAF7 interactors were identified by the MS analysis. Prominent among the complexes identified were the CCT (TriC) complex, the WASH1 complex, and STAT3 (Figure 1C; Supplementary Figures S1B,C). These interactions were predominantly cytoplasmic, although they were also detected in the nucleus. To validate these novel interactions, we performed both co-immunoprecipitation studies and proximity ligation assays (PLA).

### TAF7 interacts with the CCT (TriC) complex in the cytoplasm

The CCT complex is composed of two rings of proteins, each made up of eight different but related subunits, CCT1-CCT8 (Lopez et al., 2015). MS of TAF7-bioID biotinylated cytoplasmic proteins from TMD8 and HBL1 cells identified seven of these subunits: CCT2, CCT3, CCT4, CCT5, CCT6A, CCT7, CCT8. It is important to note that the entire complex may be brought down when only one subunit of biotinylated CCT is recovered with avidin. Thus, TAF7 interacts with the CCT complex, but may be directly interacting with only one or a few of these proteins.

**FIGURE 2:**
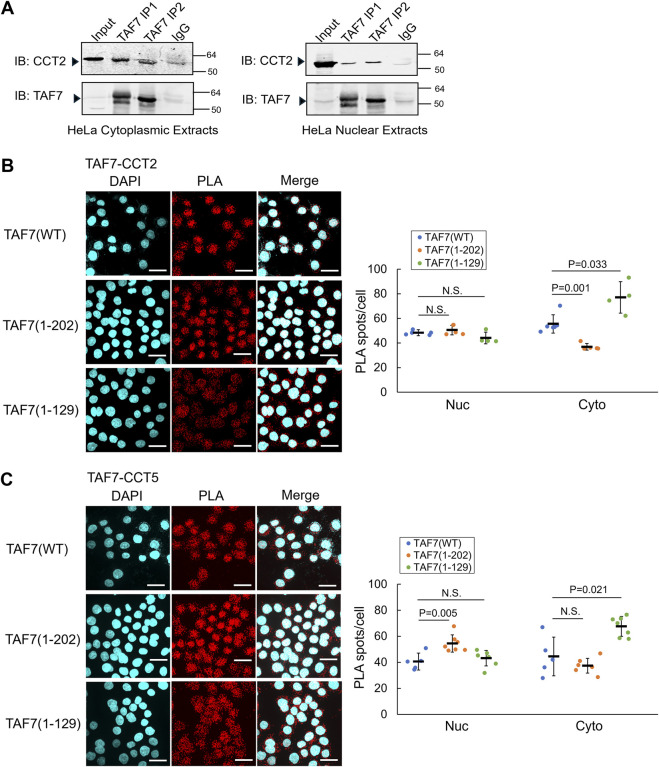
TAF7 interacts with the CCT Complex in both cytoplasm and nucleus. **(A)** Cytoplasmic (left) and nuclear (right) fractions of HeLa cell extracts were immunoprecipitated with anti-TAF7 and blotted with anti-CCT2 and anti-TAF7. Mouse Ig served as a negative control. **(B)** HeLa cells stably transfected with Flag-TAF7 WT (upper), Flag-TAF7 (1-202) (middle) and Flag-TAF7 (1-129) (bottom) were subjected to PLA analysis with anti-Flag and anti-CCT2 (left). Representative images are shown; scale bar, 25 µm. Quantitation of TAF7-CCT2 PLA spots per cell in either cytoplasm or nucleus (right). Each data point represents the average measurement from one field of view, with a total of more than 200 cells analyzed for each condition. **(C)** HeLa cells stably transfected with Flag-TAF7 WT (upper), Flag-TAF7 (1-202) (middle) and Flag-TAF7 (1-129) (bottom) were subjected to PLA analysis with anti-Flag and anti-CCT5 (left). Representative images are shown; scale bar, 25 µm. Quantitation of TAF7-CCT5 PLA spots/cell (right). Each data point represents the average measurement from one field of view, with a total of more than 200 cells analyzed for each condition.

To validate the bioID results and directly assay for the interaction of TAF7 with the CCT complex, the ability of TAF7 to co-immunoprecipitate CCT proteins from nuclear and cytoplasmic extracts of HeLa cells and MCF10A cells was assessed (Figure 2A; Supplementary Figure S2A). As shown in Figure 2A (left panel), TAF7 co-immunoprecipitates CCT2 from HeLa cytoplasmic extracts, thereby confirming the bioID MS results and demonstrating an interaction between TAF7 and CCT in the cytoplasm. Although CCT has been primarily described as a cytoplasmic complex (Frydman, 2001), we also observed co-immunoprecipitation of TAF7 and CCT2 from nuclear extracts (Figure 2A, right panel).

To determine whether TAF7 interacts with the CCT complex *in situ* in cells, PLA was performed in HeLa cells between TAF7 and CCT2 and CCT5, respectively (Figures 2B,C; Supplementary Figures S2B,C). Consistent with the bioID and co-immunoprecipitation data, complexes of TAF7 with CCT2 and with CCT5 were readily detected in the cytoplasm, as well as in the nucleus. To map the region of TAF7 that interacts with the CCT complex, PLA was performed with a set of TAF7 truncation mutants. Truncation mutants spanning aa1-202 and aa 1-129 both efficiently interact with CCT2 and CCT5, mapping the region of interaction to the amino terminus of TAF7 (Figures 2B,C).

As we have shown previously, within the 349 aa TAF7, the nuclear localization signal (NLS) and overlapping RNA binding domain are located between aa 140-155; the nuclear export signal (NES) is located between aa 202-244 (Cheng et al., 2021). The TAF7 truncation mutant spanning aa 1-202 contains the NLS, but not the NES (Cheng et al., 2021). Thus, it can enter the nucleus but not exit to the cytoplasm, Accordingly, cytoplasmic CCT complexes with TAF7 (1-202) are depleted relative to those with TAF7 (WT), suggesting that the accumulation of TAF7 (1-202) in the nucleus retains the complexes. The TAF7 truncation mutant extending from aa 1-129 contains neither the NLS or NES and is enriched in the cytoplasm (Cheng et al., 2021). Accordingly, CCT complexes with this truncation preferentially accumulate in the cytoplasm.

### TAF7 interacts with the WASH complex in the cytoplasm

**FIGURE 3:**
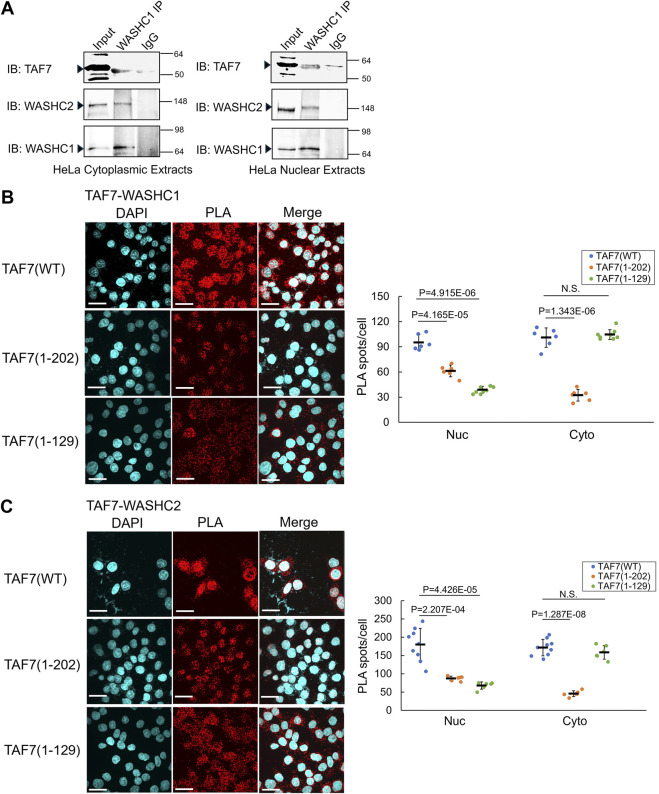
TAF7 interacts with the WASH complex in both cytoplasm and nucleus. **(A)** HeLa cytoplasmic (left) or nuclear (right) extracts were immunoprecipitated with anti-WASHC1 and immunoblotted with anti-TAF7, anti-WASHC2, and anti-WASHC1. **(B)** HeLa cells stably transfected with Flag-TAF7 WT (upper), Flag-TAF7 (1-202) (middle) and Flag-TAF7 (1-129) (bottom) were subjected to PLA analysis with anti-Flag and anti-WASHC1 (left). Representative images are shown; scale bar, 25 µm. Quantitation of TAF7-WASHC1 PLA spots per cell in either cytoplasm or nucleus (right). Each data point represents the average measurement from one field of view, with a total of more than 200 cells analyzed for each condition. **(C)** HeLa cells stably transfected with Flag-TAF7 WT (upper), Flag-TAF7 (1-202) (middle) and Flag-TAF7 (1-129) (bottom) were subjected to PLA analysis with anti-Flag and anti-WASHC2 (left). Representative images are shown; scale bar, 25 µm. Quantitation of TAF7-WASHC2 PLA spots/cell (right). Each data point represents the average measurement from one field of view, with a total of more than 200 cells analyzed for each condition.

The WASH complex is composed of five distinct proteins, WASHC1 (WASH1), WASHC2A/C (FAM21 A/C), WASHC3 (CCDC53), WASHC4 (KIAA1033, SWIP), and WASHC5 (KIAA0196, Strumpellin) (Derivery et al., 2009; Gomez and Billadeau, 2009; Jia et al., 2010). Of these, all but WASHC4 were detected in the MS analysis of cytoplasmic fractions following TAF7 bioID pull-down (Figure 1C; Supplementary Figures S1B,C). To validate this interaction experimentally, we performed both co-immunoprecipitation experiments and PLA. As shown in Figure 3A, WASH can co‐immunoprecipitate TAF7 from cytoplasmic (left panel), as well as nuclear extracts (right panel) of HeLa cells. The interaction of TAF7 with the WASHC1 complex is readily detectable by PLA in HeLa cells, with both WASHC1 or WASHC2 (Figures 3B,C; Supplementary Figure S3). In both cases, complexes are observed in the cytoplasm and the nucleus, distributed nearly equally.

The TAF7 truncation mutants mapped the interacting domain of TAF7 to a region in the N terminus, between aa 1 and 129 (Figures 3B,C). These findings demonstrate an interaction between the N terminal domain of TAF7 and the WASH complex. In contrast to its interaction with CCT, the TAF7 1-202 interactions with the WASH complex are decreased in both the nucleus and the cytoplasm.

### TAF7 interacts with STAT3 in the cytoplasm

**FIGURE 4:**
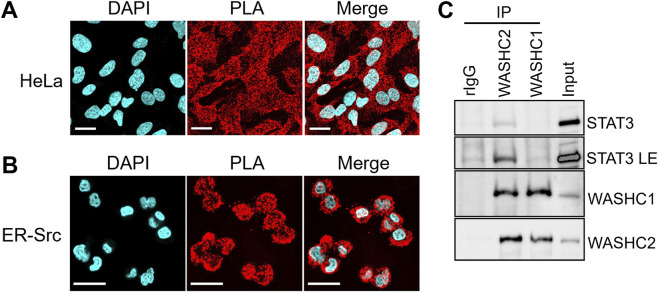
TAF7 interacts with STAT3 in both cytoplasm and nucleus. **(A)** HeLa cells were subjected to PLA analysis with anti-TAF7 and anti-STAT3. Representative images are shown; scale bar, 25 µm. **(B)** MCF10A-ER-Src cells were subjected to PLA analysis with anti-TAF7 and anti-STAT3. Representative images are shown; scale bar, 25 µm. **(C)** Cell extracts were immunoprecipitated with anti-WASHC1 and anti-WASHC2 antibodies and blotted with anti-STAT3, anti-WASHC1, anti-WASHC2 antibodies. Rabbit Ig served as a negative control. LE: longer exposure.

To validate the bioID finding that STAT3 interacts with TAF7 in the cytoplasm, we performed PLA of TAF7 and STAT3 in HeLa cells where TAF7 clearly co-localizes with STAT3 (Figure 4A; Supplementary Figure S4B). In addition, STAT3 can co-immunoprecipitate TAF7 from cytoplasmic (left panel), as well as nuclear, extracts (right panel) of HeLa cells (Supplementary Figure S4A). We also examined the interaction of TAF7 with STAT3 in MCF10A-ER-Src cells (Figure 4B). MCF10A cells are a normal epithelial breast cancer line that can be induced to transform by the kinase Src. MCF10A-ER-Src cells have been stably transformed with an ER-Src construct that is active in the presence of tamoxifen (Iliopoulos et al., 2009). In the absence of tamoxifen, MCF10A-ER-Src cells are phenotypically normal. Under these conditions, TAF7 colocalizes with STAT3; complexes were found primarily in the cytoplasm (Figure 4B).

STAT3 is known to also interact with CCT (Kasembeli et al., 2014; Vallin et al., 2021). Since TAF7 interacts with STAT3, CCT and WASH, we considered the possibility that they form a multimeric complex. To assess that possibility, we next asked whether STAT3 interacted with WASH. Indeed, STAT3 is co-immunoprecipitated from HeLa cell extracts by anti-WASH antibodies directed against WASHC2A/C (Figure 4C). Taken together, these results suggest that cytoplasmic TAF7 exists in a supercomplex with STAT3, CCT and WASH.

### Cytoplasmic TAF7 levels correlate with cellular oncogenicity

**FIGURE 5:**
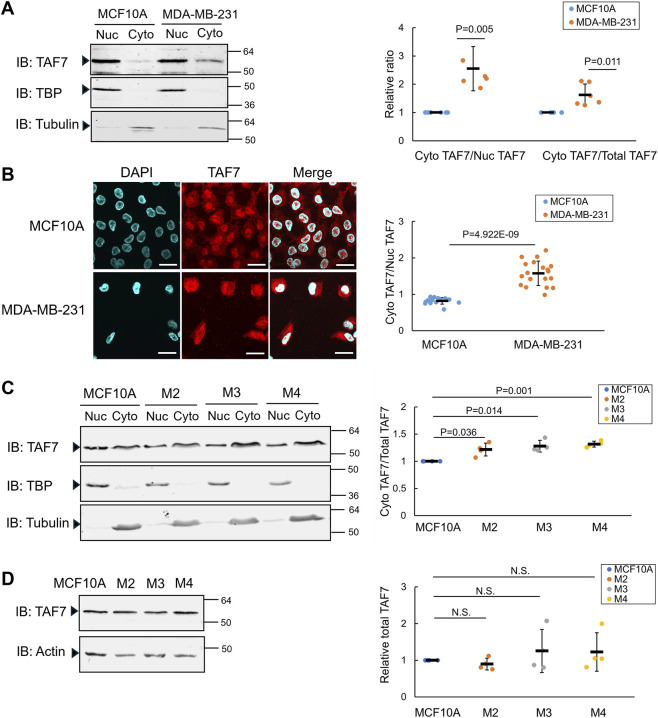
Cytoplasmic TAF7 levels correlate with tumorigenicity. **(A)** Cytoplasmic levels of TAF7 are greater in MDA-MB-231 breast cancer cells than in the non-transformed MCF10A breast cell line. Nuclear and cytoplasmic fractions were isolated from both cell types and analyzed by immunoblotting with anti-TAF7 and with anti-TBP, which served both for normalization and as a control for cytoplasmic contamination with nuclear proteins and with anti-tubulin which similarly served as a control for both normalization and for nuclear contamination with cytoplasmic proteins (left). Quantitation of distribution of TAF7 between the cytoplasm and nucleus in MDA-MB-231 and MCF10A cells (right). The distribution is calculated with as a cytoplasmic/nuclear ratio or as a fraction of TAF7 in the cytoplasm. Both calculations were corrected for TBP and tubulin levels and cross contamination. Relative levels of nuclear and cytoplasmic TAF7 were determined by densitometric analysis of six independent fractionations. **(B)** Immunofluorescence of MDA-MB-231 and MCF10A cells stained with anti-TAF7 and DAPI (left). Representative images are shown; scale bar, 25 µm. The relative level of cytoplasmic TAF7 increases with pathogenicity of cancer cell lines (right). Quantitation of the distribution of TAF7 between the cytoplasm and nucleus as determined from immunofluorescence. Each data point represents the average measurement from one field of view, with a total of more than 200 cells analyzed for each condition. **(C)** The sequential series of breast cancer cell lines, derived from the normal MCF10A breast cell line, were fractionated into nuclear and cytoplasmic compartments (left). MCF10A, normal breast cell line; M2, pre-neoplastic; M3 low-grade; M4 high grade. The levels TAF7 were probed by immunoblotting with anti-TAF7 antibody; anti-TBP and anti-tubulin served as controls for contamination of the cytoplasmic and nuclear compartments, respectively. The relative levels of cytoplasmic TAF7 were determined from densitometric analysis, correcting for TBP and tubulin. The data represents four independent experiments (right). **(D)** Total cellular TAF7 levels do not change with increased pathogenicity (left). Total cell extracts of MCF10A and the M2-M4 cell lines were immunoblotted with TAF7 (upper). Total TAF7 levels were determined by densitometric analysis of four independent experiments (right).

Since STAT3, CCT and WASH have all been implicated in cancer, we hypothesized that cytoplasmic TAF7 might also correlate with cellular transformation. Consistent with this hypothesis, the relative levels of cytoplasmic TAF7 in the highly pathogenic breast cancer cell line, MDA-MD-231, are about 2.5 times greater than in the non-transformed MCF-10A line, as measured either by immunoblotting or immunofluorescence (Figures 5A,B). A similar striking difference in cytoplasmic TAF7 levels is observed between mouse T cell lymphoma cells and primary T cells (Supplementary Figures S5A,B). These results suggested that cytoplasmic TAF7 increases with cellular transformation.

### Relative levels of cytoplasmic TAF7 increase with increasing pathogenicity of cell lines

To further explore the correlation between cytoplasmic TAF7 levels and cellular transformation, we examined a series of cell lines with varying degrees of pathogenicity. Mouse embryo fibroblasts (MEF), the human kidney (HEK293) and the breast MCF10A cell lines are phenotypically normal and non-tumorigenic. MCF7 and MDA231 are breast cancer lines, with MDA231 being highly pathogenic. HeLa cells derive from a highly aggressive ovarian cancer. As shown in Supplementary Figure S5C, tumorigenic lines have higher relative cytoplasmic TAF7 levels compared with the normal cell lines, consistent with the hypothesis that cytoplasmic TAF7 levels scale with oncogenicity.

To assess the possibility that TAF7 cytoplasmic levels increase as a function of cellular transformation, we examined a series of breast cancer cell lines derived sequentially from the normal MCF10A progenitor line, which display increasing degrees of pathogenicity (Tang et al., 2003). The M2 line was generated by transfecting MCF10A cells with H-Ras (Dawson et al., 1996). It is preneoplastic as defined by its altered morphology, but unable to initiate tumors in mice. Serial passage of M2 as a xenograft resulted in a line with low grade tumorigenicity (M3) (Santner et al., 2001). Finally, continued serial passage of M2 xenografts generated a cell line with high tumorigenic potential (M4) (Santner et al., 2001). Analysis of the cellular distribution of TAF7 revealed that the relative level of cytoplasmic TAF7 increased in the breast cancer cell lines with increasing pathogenicity (Figure 5C). Thus, the M2 line already displays modestly increased cytoplasmic TAF7 while the M3 and M4 lines have significantly higher cytoplasmic levels, relative to parental MCF10A cells which have an approximately equal distribution of TAF7 between cytoplasm and nucleus. These increases are not simply due to increased synthesis of TAF7, since total cellular TAF7 did not differ significantly among the four breast cancer lines (Figure 5D).

### MYC-induced T-cell acute lymphoblastic leukemia induces increased cytoplasmic TAF7 levels

**FIGURE 6:**
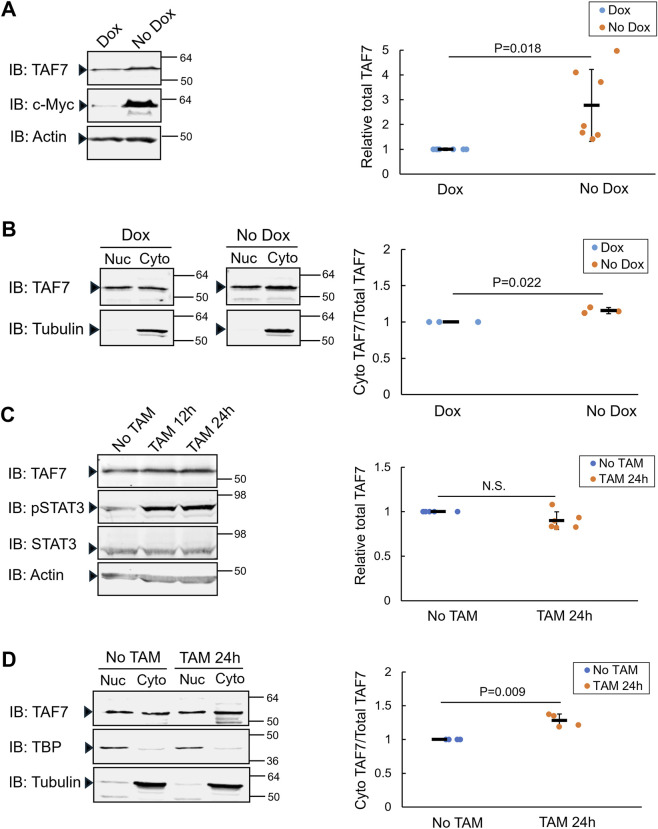
Relative levels of cytoplasmic TAF7 increase during cell transformation. **(A)** Murine T-ALL cells, which remain transformed in the absence of Dox, were treated with Dox for 24 h, after which total TAF7, MYC and actin levels were assessed by immunoblotting (left). Relative levels of TAF7 in the transformed cells were determined from densitometry, corrected for actin, and normalized to the dox-treated normal cells (right). The data represents seven independent experiments. **(B)** T-ALL cells, treated or not with Dox, were fractionated into nuclear and cytoplasmic extracts and probed with anti-TAF7 and anti-tubulin (left). The relative levels of cytoplasmic TAF7 were determined by densitometry, corrected for tubulin (right). The data represents three independent experiments. **(C)** Extracts from MCF10A‐ER‐Src cells, treated for various times with tamoxifen, were immunblotted with anti-TAF7, anti-phoshpoSTAT3 and anti-STAT3 (left). Cellular levels of TAF7 were determined from densitometry and represent the results of six independent experiments (right). **(D)** MCF10A-ER-Src cells, treated or not with tamoxifen, were fractionated into nuclear and cytoplasmic components and probed with anti-TAF7, anti-TBP and anti-tubulin (left). Relative levels of cytoplasmic TAF7 were determined from densitometric analysis after correcting for cytoplasmic (tubulin) and nuclear contamination (TBP). Results are calculated as cytoplasmic TAF7/total TAF7 (right) and represent the results from four independent experiments.

To examine whether increases in cytoplasmic TAF7 are increased concomitant with transformation, we turned to a model in which overexpression of the oncogene Myc in hematopoietic cells leads to leukemic transformation. In this murine transgenic model system, in which Myc expression is driven by a Tet-off Eμ promoter, mice develop lymphoid and myeloid tumors (Felsher and Bishop, 1999). Leukemic cell lines derived from these mice express high levels of MYC and maintain their leukemic phenotype in the absence of doxycycline (Figure 6A). However, the addition of doxycycline represses expression of MYC and cells lose their tumorigenicity. This model provided us with an approach to directly assess the correlation between cytoplasmic TAF7 and cellular transformation.

In the presence of doxycycline, which suppresses transformation, cells express comparable levels of TAF7 in the cytoplasm and nucleus (Figure 6B). Removal of Dox results in cell transformation and is accompanied by a significant increase in the relative levels of cytoplasmic TAF7 (Figure 6B). In contrast to the breast cancer cell lines, where total TAF7 levels do not increase with increasing pathogenicity, the removal of Dox increases both MYC and total TAF7 levels in the leukemic cells (Figure 6A). Nevertheless, these results demonstrate a direct correlation between cellular oncogenicity and the fraction of TAF7 in the cytoplasm, independent of the total levels of TAF7.

### Cytoplasmic TAF7 levels increase with ER-Src induced breast cancer cell transformation

As noted above, treatment of MCF10A-ER-Src cells with tamoxifen results in phenotypic cellular transformation within 24 h. Transformation is accompanied by phosphorylation of the transcription factor STAT3 (Iliopoulos et al., 2009). Using this system, we can also examine the effect of transformation on TAF7 localization within the cell. In the absence of tamoxifen, cells look phenotypically normal (Supplementary Figure S6, left). Following treatment with tamoxifen, cells are phenotypically transformed and STAT3 phosphorylation increases; total TAF7 levels are not significantly altered (Figure 6C; Supplementary Figure S6). Importantly, there is a significant increase in the relative abundance of cytoplasmic TAF7 (Figure 6D).

Together, these analyses document a direct correlation between cellular transformation and cytoplasmic TAF7 localization in several different cell lines and systems.

### TAF7 increasingly colocalizes with STAT3 during cellular transformation of MCF10A cells by Src

**FIGURE 7:**
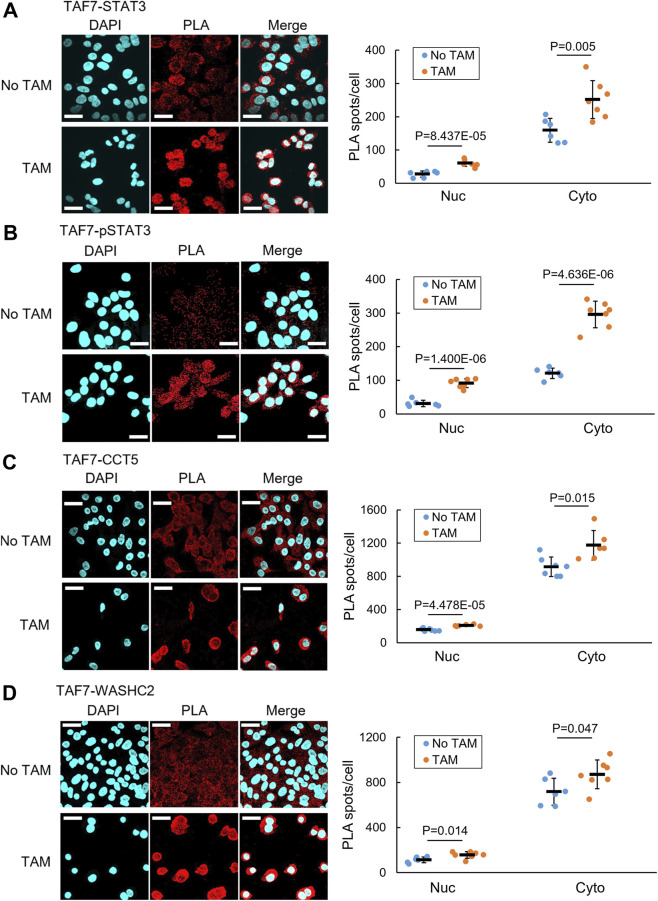
TAF7 co-localization with STAT3 in both nucleus and cytoplasm increases during cellular transformation. Er-Src MCF10A cells were cultured without (upper) or with (lower) tamoxifen for 24 h and then assayed by PLA for the interaction of TAF7 with STAT3 **(A)**, p-STAT3 **(B)**, CCT5 **(C)**, FAM21 **(D)**. Immunofluorescence, left; Representative images are shown; scale bar, 25 µm. PLA spots/cells in the nucleus and cytoplasm were quantitated (right). Each data point represents the average measurement from one field of view, with a total of more than 200 cells analyzed for each condition.

Having found that cytoplasmic TAF7 and pSTAT3 each increase during MCF10A-ErScr transformation, we next asked whether the interaction between TAF7 and STAT3 also increases during transformation, as assessed by PLA. Indeed, there is a significant increase in both TAF7/STAT3 and TAF7/pSTAT3 complexes following tamoxifen treatment in both the cytoplasm and the nucleus (Figures 7A,B; Supplementary Figure S7). Thus, Src-mediated transformation of MCF10A cells is accompanied by increased cytoplasmic TAF7, increased phosphoSTAT3, and increased association of TAF7 with both STAT3 and phosphoSTAT3.

### TAF7’s interaction with both WASH and CCT increases during transformation

Like STAT3, both WASH and CCT complexes have been implicated in oncogenesis (Yu et al., 2009; Zech et al., 2011; Deng et al., 2015; Huang et al., 2017; Vallin and Grantham, 2019; Zou et al., 2020; Ghozlan et al., 2022; Zeng et al., 2024). Therefore, we next asked whether Src-mediated transformation of MCF10A cells similarly induced increased interactions of TAF7 with either WASH or CCT complexes, as assessed by PLA. TAF7 colocalized robustly with the CCT component CCT5 in the untreated MCF10A cells, like its interaction in HeLa cells (Figure 7C). Following induction of transformation, the interaction between TAF7 and the CCT complex increased significantly (Figure 7C). The interactions were primarily localized to the cytoplasm, consistent with the known localization of CCT and with the increased cytoplasmic TAF7 following transformation. Similarly, TAF7 colocalized with the WASH component, WASHC2, in the non-transformed MCF10A-ER-Src cells (Figure 7D). Like the interactions with STAT3 and CCT, transformation of the cells with tamoxifen significantly increased TAF7’s colocalization with the WASH complex (Figure 7D).

Taken together, these results indicate that cellular transformation is associated with the increased interaction of TAF7 with its partners.

## Discussion

TAF7 was originally identified as a passive component of the general transcription factor TFIID; however, our previous studies established that TAF7 plays active regulatory roles across multiple stages of gene expression (Gegonne et al., 2006; Cheng et al., 2021). Specifically, TAF7 functions as a regulator of transcription initiation and elongation, acts as an RNA chaperone by binding CUG motifs within the 3′ UTR of target transcripts and directing their transport from the nucleus to cytoplasmic polysomes, and modulating translation. These findings indicate that, although it was originally characterized exclusively as a nuclear factor, TAF7 also executes essential cytoplasmic functions. The present study remarkably further expands this multi-functional repertoire by identifying a transformation-associated cytoplasmic TAF7 interactome that includes STAT3, the endosomal sorting WASH complex, and the protein-folding CCT complex. Notably, cellular transformation was accompanied by increased relative cytoplasmic TAF7 levels and enhanced association with phospho-STAT3, WASH, and CCT—factors with well-established roles in cancer—linking cytoplasmic TAF7 to oncogenic signaling and proteostatic pathways.

**FIGURE 8:**
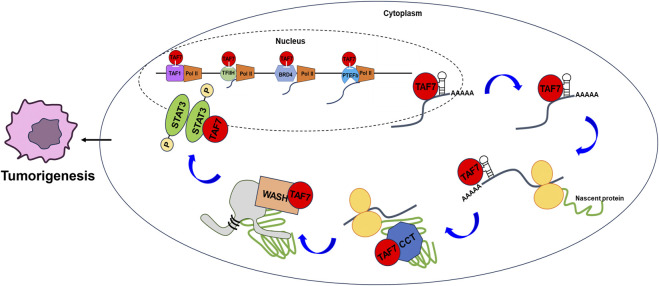
Model for TAF7 coordination of gene expression. TAF7 regulates transcription intitiation, pause release and elongation through its interactions with TFIID, TFIIH, BRD4 and CDK9 respectively. TAF7 binds to the 3′ UTR of target transcripts, transporting them to cytoplasmic polysomes to regulate translation, interacting with CCT to ensure proper protein folding and with the WASH complex for proper protein sorting. TAF7 interacts with STAT3, potentially translocating together to the nucleus to regulate transcription.

Based on these findings, we propose a model in which TAF7 is a pleiotropic controller of gene expression: It links the processes of transcription, mRNA export, translation, protein folding and endosomal sorting (Figure 8). In normal cells, TAF7 functions to maintain cellular homeostasis through its coordination of processes involved across the spectrum of functions necessary for gene expression. However, oncogenic transformation perturbs this homeostasis, resulting in a redistribution of TAF7 from the nucleus to the cytoplasm, where it acquires context-dependent functions that support transformation. The association of TAF7 with activated STAT3 may facilitate sustained oncogenic signaling. Interactions with the WASH complex could couple endosomal trafficking with the spatial regulation of signaling and RNA transport. The interaction of TAF7 with the CCT chaperonin complex may promote folding and stability of newly synthesized or oncogenic proteins, thereby supporting the increased proteostatic demand of transformed cells. Together, these interactions suggest that cytoplasmic TAF7 functions as a control node in both normal and transformed cells that integrates transcription-derived outputs with post-transcriptional and post-translational pathways.

The presence of TAF7 in the cytoplasm led us to ask how widespread its cytoplasmic localization is across different cell lines. Surprisingly, we observed cytoplasmic levels of TAF7 in every cell line we examined, with the lowest relative levels in non-transformed cells and the highest relative levels in transformed cells. Particularly intriguing was the finding that the relative levels of cytoplasmic TAF7 increased with increasing pathogenicity of the cancer cell line, whether the total level of TAF7 increased or not. Analysis of cellular transformation in three distinct models - breast cancer cell lines, induced T-ALL transformation and Src-induced transformation of a normal breast cell line–provided further evidence that cytoplasmic TAF7 levels increase during transformation. Thus, there is a direct correlation between pathogenicity and cytoplasmic TAF7.

Consistent with our findings, analysis of patient data has reported that TAF7 is part of a risk signature in HNSCC and its high expression negatively correlated with survival in TNBC (Pang et al., 2025; Zhang et al., 2025). Furthermore, although mutations in TAF7 are uncommon, analysis of TCGA data reveals that cancer patients with TAF7 mutations have better overall survival across 15 cancer types. In uterine cancer, progression free survival is significantly better in patients with TAF7 mutations (Supplementary Figure S8). Although none of these analyses examined the cellular localization of TAF7 in the cancer cells, these studies document a role for TAF7 in cancer.

Insights into the potential functions of cytoplasmic TAF7 in both normal and transformed cells were gained by identifying cytoplasmic proteins with which TAF7 interacts, none of which had been previously described. Among the interactors was STAT3, a transcription factor whose function is tightly regulated by its cellular localization (Levy and Darnell, 2002). In the absence of signaling, STAT3 is primarily in the cytoplasm. Following external receptor signaling, it is phosphorylated by Src causing it to translocate into the nucleus to activate transcription of target genes. In non-transformed cells, the TAF7 interaction with STAT3 occurs primarily in the cytoplasm. Following Src-mediated transformation of MCF10A cells, the interaction of TAF7 with STAT3 increased in parallel with STAT3 phosphorylation, in both the cytoplasm and the nucleus. This suggests that TAF7 and STAT3 may co-translocate to the nucleus to coordinately promote transcription.

Proper folding of nascent peptides depends on the co-translational activity of protein chaperones (Frydman, 2001). Among them is CCT, a member of the chaperonin family of multiprotein complexes that hydrolyze ATP during the folding of their substrates (Lopez et al., 2015). CCT forms a cylindrical barrel through which most of the folding occurs (Spiess et al., 2004). Surprisingly, it also folds proteins too large to fit within the barrel by a sequential folding process (Rüssmann et al., 2012). TAF7 interacted with CCT primarily in the cytoplasm, although some nuclear co-localization occurs. This interaction increased in transformed cells. Interestingly CCT is upregulated in several cancers, including nasopharyngeal and neuroblastoma, leading to the hypothesis that cancers become “addicted” to chaperones (Cox et al., 2022; Ghozlan et al., 2022; Wu and Peng, 2024). Whether or how TAF7 affects CCT function remains to be determined. We cannot rule out the possibility that the interaction of TAF7 with CCT reflects CCT-mediated folding. However, the mapping of the interaction to the N-terminal region of TAF7 makes this less likely. Rather, and consistent with our previous findings that TAF7 is associated with polysomes, we speculate that TAF7 may recruit CCT to polysomes, to facilitate co-translational folding.

Endosomal protein sorting is critical for a variety of cellular functions to maintain normal homeostasis. The pentameric WASH complex, plays a pivotal role in intracellular trafficking. It is localized mainly to endosomes but can be detected throughout the cell including the nucleus. Indeed, the WASHC2 subunit interacts with the transcription factor NFκB in the nucleus of pancreatic cells where it is chromatin bound (Deng et al., 2015). Depletion of WASHC2 sensitizes pancreatic cancer cells to apoptosis, suggesting a role for WASHC2 and possibly the WASH complex in cancer (Deng et al., 2015). TAF7 complexes with WASH were observed in both the cytoplasm and the nucleus of non-transformed cells. These complexes increased during transformation, suggesting that both factors contribute to maintaining homeostasis of transformed cells. It is also possible that the interaction with TAF7 serves to coordinate WASH complex activities with other cellular activities, to maintain homeostasis in normal and transformed cells.

These findings lead to the question of how a relatively small molecule of 55 kD is able to interact with so many different proteins. The C-terminal half of TAF7 is intrinsically disordered, presumably allowing it to undergo “induced-fit” conformational changes that enable interactions with its disparate partners. This multiplicity of interactions is not unprecedented. The 49 kD MYC protein has been reported to interact with over 50 different proteins (Kalkat et al., 2018). We postulate that this multiplicity of interactions reflects the functional pleiotropy of TAF7.

The role of TAF7 in tumorigenesis remains to be firmly established. Although the present studies demonstrate a correlation between relative levels of cytoplasmic TAF7 and tumorigenesis, whether increased cytoplasmic TAF7 contributes to transformation or is a consequence remains to be determined. Future experiments will address this question and further test the model that TAF7 functions as an integration node to maintain cellular homeostasis in normal and transformed cells.

## Data Availability

Data are deposited on MassIVE DB with the identifier PXD080431: http://massive.ucsd.edu/ProteoSAFe/status.jsp?task=eadfaf76b17c46bebc39e421bfbd0c4d.
