# Supplementary material for: TAF7 accumulates in the cytoplasm during cellular transformation and engages STAT3, WASH, and CCT
Source: Front Cell Dev Biol. 2026 Jul 8;14:1855716. doi: 10.3389/fcell.2026.1855716 (PMC13388811; doi:10.3389/fcell.2026.1855716)
Supplement: Supplementary file 1 [file DataSheet1.docx]

**A**


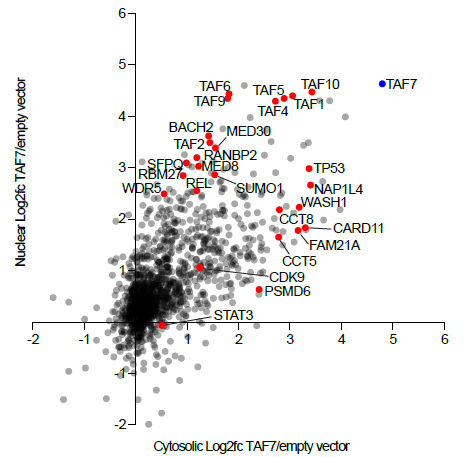


**B**


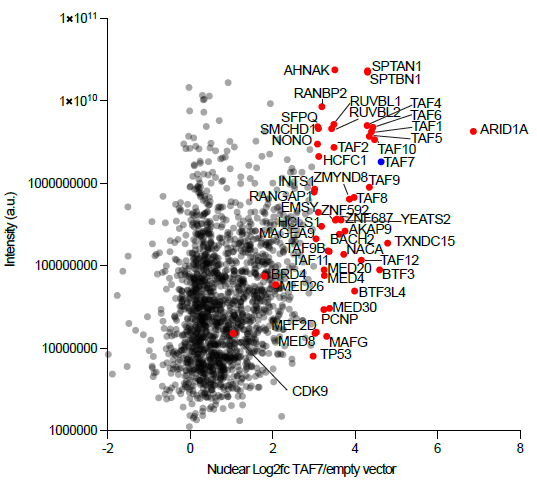


**C**


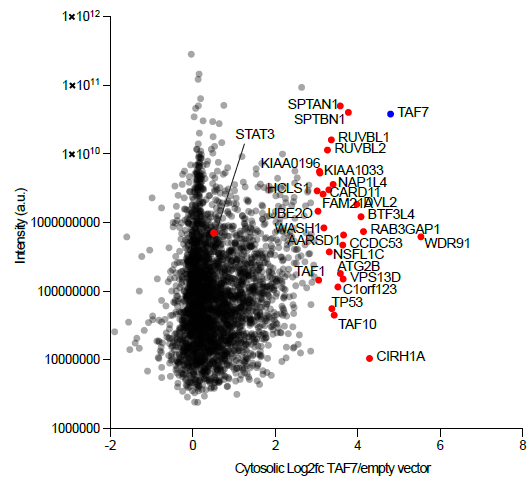


Figure S1. BioID probing identifies STAT3, WASH and CCT as TAF7 interactors in the cytoplasm.

1. Scatter plot of essential nuclear interactome of TAF7.
2. Scatter plot of essential cytoplasmic interactome of TAF7.
3. Scatter plot comparing nuclear and cytoplasmic interactome of TAF7.


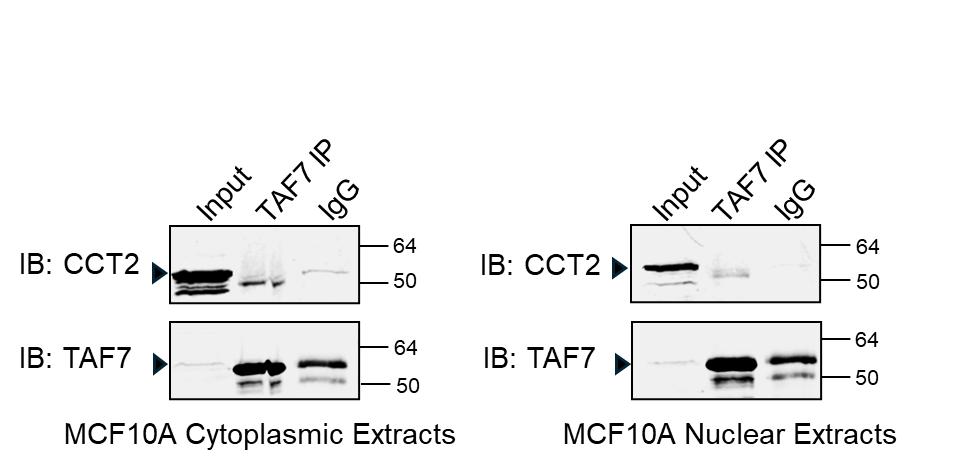


**A**

**B**


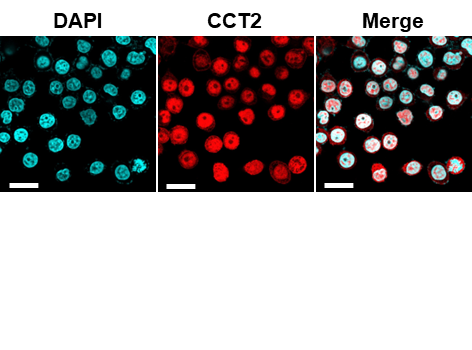

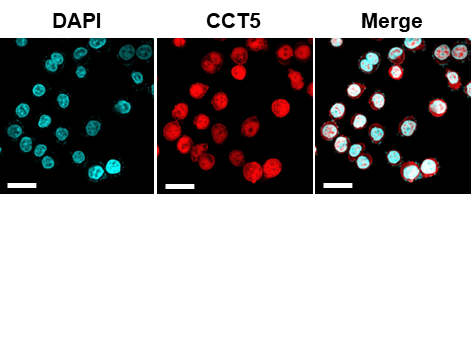


**C**

**Figure S2. TAF7 interacts with the CCT complex in both cytoplasm and nucleus.**

1. Cytoplasmic (left) and nuclear (right) fractions of MCF10A cell extracts were immunoprecipitated with anti-TAF7 and blotted with anti-CCT2 and anti-TAF7. Mouse Ig served as a negative control.
2. Immunofluorescence of HeLa cells stained with anti-CCT2 and DAPI. Representative images are shown; scale bar, 25 µm.
3. Immunofluorescence of HeLa cells stained with anti-CCT5 and DAPI. Representative images are shown; scale bar, 25 µm.

**A**


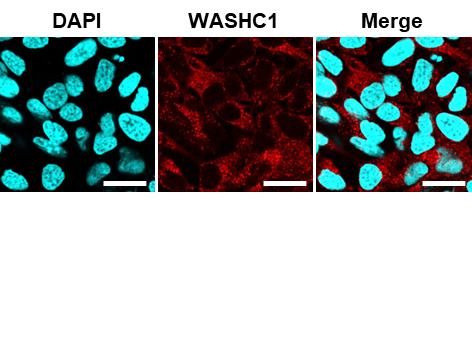


**B**


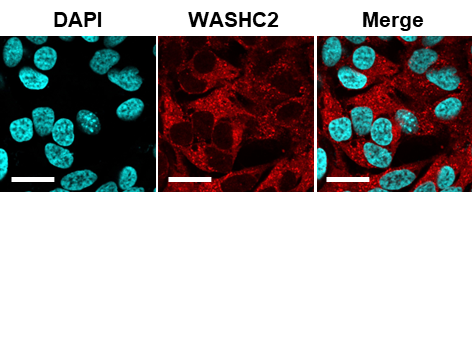


**Figure S3. WASH complex in HeLa cells is primarily cytoplasmic.**

1. Immunofluorescence of HeLa cells stained with anti-WASHC1 alone and DAPI. Representative images are shown; scale bar, 25 µm.
2. Immunofluorescence of HeLa cells stained with anti-WASHC2 alone and DAPI. Representative images are shown; scale bar, 25 µm.


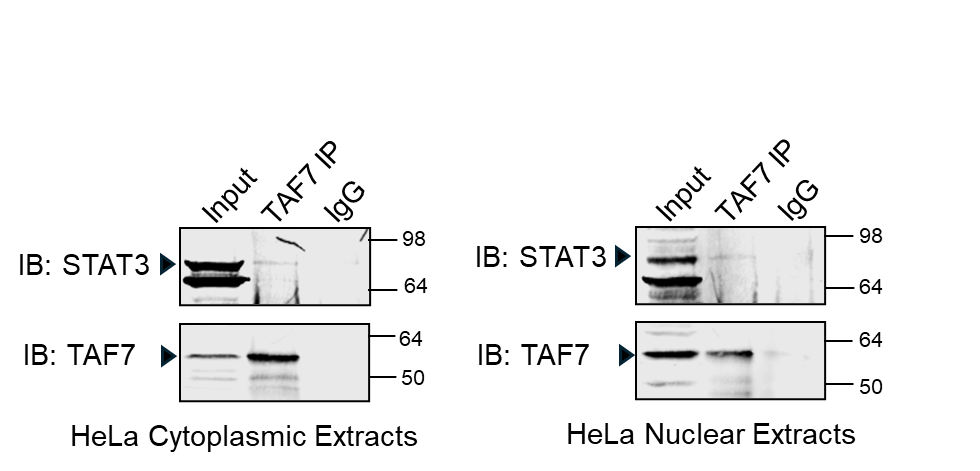


**A**

**B**


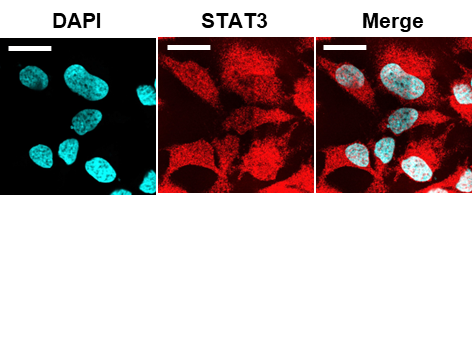


**Figure S4. TAF7 interacts with STAT3 in both cytoplasm and nucleus.**

1. Cytoplasmic (left) and nuclear (right) fractions of HeLa cell extracts were immunoprecipitated with anti-TAF7 and blotted with anti-STAT3 and anti-TAF7. Mouse Ig served as a negative control.
2. Immunofluorescence of HeLa cells stained with anti-STAT3 alone and DAPI. Representative images are shown; scale bar, 25 µm.

**A**


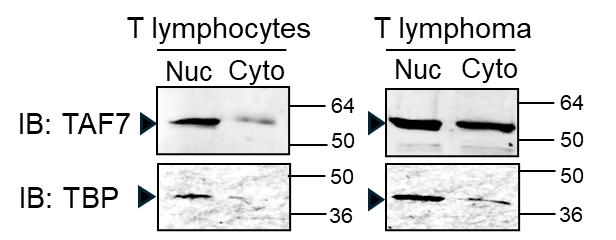

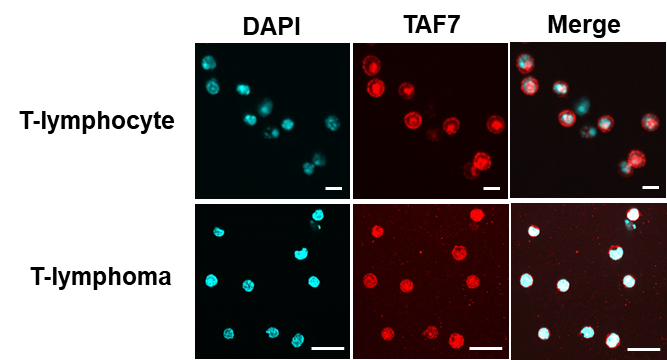


**B**


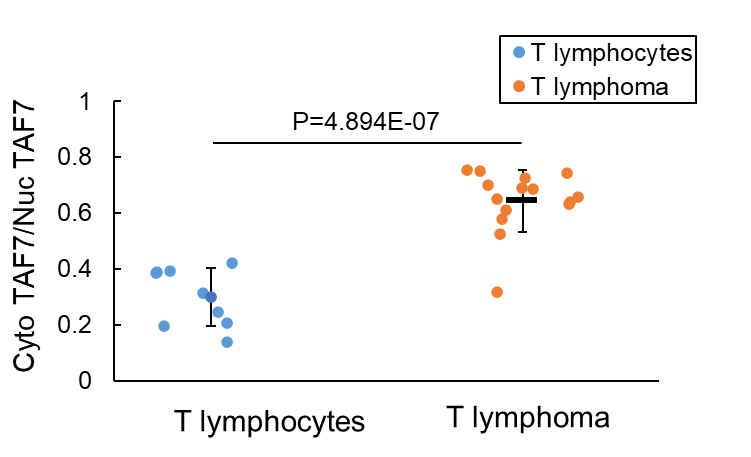


**C**


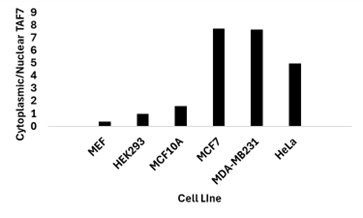


**Figure S5. Cytoplasmic TAF7 levels correlate with tumorigenicity.**

1. Cytoplasmic levels of TAF7 are greater in T-cell lymphoma than in the non-transformed T-lymphocytes. Nuclear and cytoplasmic fractions were isolated from both cell types and analyzed by immunoblotting with anti-TAF7 and with anti-TBP, which served both for normalization and as a control for cytoplasmic contamination with nuclear proteins.
2. Immunofluorescence of T-cell lymphoma and T-lymphocytes stained with anti-TAF7 and DAPI (left). Representative images are shown; scale bar, 25 µm. Quantitation of the distribution of TAF7 between the cytoplasm and nucleus as determined from immunofluorescence (right). Each data point represents the average measurement from one field of view, with a total of more than 200 cells analyzed for each condition.
3. The relative level of cytoplasmic TAF7 increases with pathogenicity of cancer cell lines.

**No TAM**

**TAM**


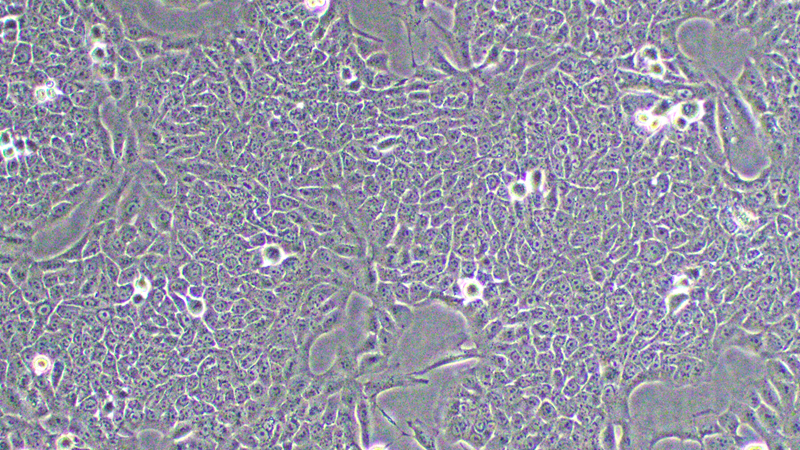

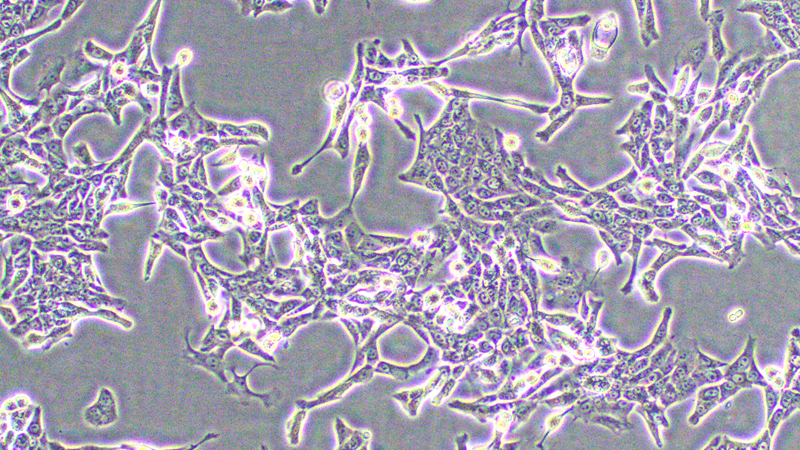


**Figure S6. Tamoxifen treatment of ER-Src transformed MCF10A cells induces cell transformation.** ER-Src transformed MCF10A cells were treated (TAM) or not (no TAM) with tamoxifen for 24 hours, following which photomicrographs of the cells were taken.


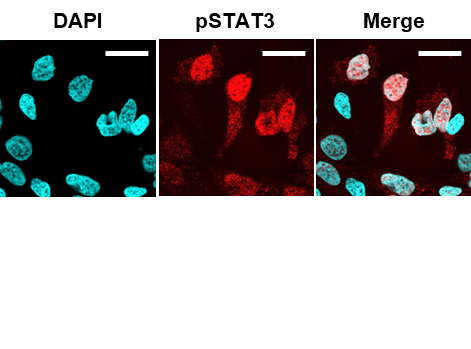


**Figure S7. Phospho-STAT3 is primarily nuclear in HeLa cells.** Immunofluorescence of HeLa cells stained with anti-pSTAT3 alone and DAPI. Representative images are shown; scale bar, 25 µm.

**A**

**B**


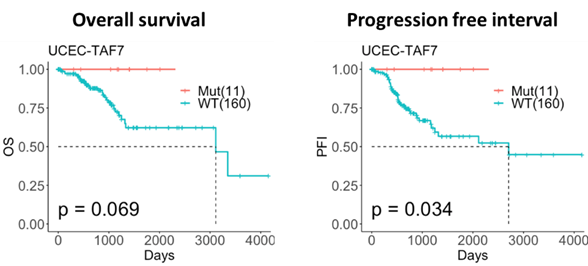

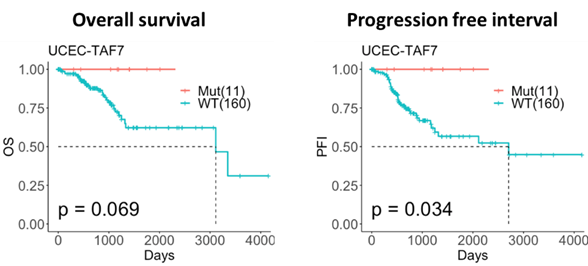


**Figure S8. Survival analysis of TCGA-UCEC patients by TAF7 mutation status.** Kaplan–Meier curves illustrate that patients with TAF7 mutations (Mut, red; n = 11) exhibited a trend toward improved overall survival **(A)** and progression-free interval **(B)** compared to wild-type patients (WT, blue; n = 160). Dashed lines indicate median survival. Statistical significance was determined by the log-rank test. Somatic mutation data and clinical metadata for the Uterine Corpus Endometrial Carcinoma (UCEC) cohort were retrieved from The Cancer Genome Atlas (TCGA) data portal (<https://portal.gdc.cancer.gov/>). All statistical analyses were performed on R version 4.5.2. Due to the small number of patients with TAF7 mutation, a P-value < 0.1 was deemed significant.
